# Emerging Cancer Epigenetic Mechanisms Regulated by All-Trans Retinoic Acid

**DOI:** 10.3390/cancers12082275

**Published:** 2020-08-14

**Authors:** Stefano Rossetti, Nicoletta Sacchi

**Affiliations:** Department of Cancer Genetics and Genomics, Roswell Park Comprehensive Cancer Center, Buffalo, NY 14263, USA; stefano.rossetti@roswellpark.org

**Keywords:** all-trans retinoic acid (RA), RA receptor α (RARA), annexins, microRNAs, cancer

## Abstract

All-trans retinoic acid (RA), which is the dietary bioactive derivative obtained from animal (retinol) and plant sources (beta-carotene), is a physiological lipid signal of both embryonic and postembryonic development. During pregnancy, either RA deficiency or an excessive RA intake is teratogenic. Too low or too high RA affects not only prenatal, but also postnatal, developmental processes such as myelopoiesis and mammary gland morphogenesis. In this review, we mostly focus on emerging RA-regulated epigenetic mechanisms involving RA receptor alpha (RARA) and Annexin A8 (ANXA8), which is a member of the Annexin family, as well as ANXA8 regulatory microRNAs (miRNAs). The first cancer showing ANXA8 upregulation was reported in acute promyelocytic leukemia (APL), which induces the differentiation arrest of promyelocytes due to defective RA signaling caused by RARA fusion genes as the PML-RARA gene. Over the years, ANXA8 has also been found to be upregulated in other cancers, even in the absence of RARA fusion genes. Mechanistic studies on human mammary cells and mammary glands of mice showed that ANXA8 upregulation is caused by genetic mutations affecting RARA functions. Although not all of the underlying mechanisms of ANXA8 upregulation have been elucidated, the interdependence of RA-RARA and ANXA8 seems to play a relevant role in some normal and tumorigenic settings.

## 1. Introduction

Historically, Egyptians, the Greek scholar Hippocrates, and Chinese Medicine used liver to treat night blindness for thousands of years. However, they did not know that liver is a source of vitamin A [1]. Preformed vitamin A (retinol and its esterified form, retinyl esters) in foods from animal sources and provitamin A (beta-carotene) in food from plant sources are metabolized intracellularly into all-trans retinoic acid (RA), which is a morphogen and lipid signal that governs developmental processes throughout a human’s life [2,3,4,5,6].

A proper dietary vitamin A (retinol) level is indispensable for embryonic and fetal development and during one’s entire life [7]. However, vitamin A deficiency during infancy, childhood, pregnancy, and lactation increases the risk of several diseases, including night blindness (xerophthalmia), anemia, and gastrointestinal disorders [3,4,6,8,9,10,11,12,13,14]. Anomalies of embryonic and fetal development are also induced by an excessive vitamin A intake. These observations were first found in a rat model by Dr. Sidney Cohlan in 1953 [15].

The discovery of vitamin A’s teratogenicity was a breakthrough showing that an excess or deficiency of dietary vitamin A in pregnant women adversely affected fetal development. Over the years, Cohlan’s work prompted hundreds of other experiments examining how excessive vitamin A and its metabolites, including RA, adversely impacted fetal development. Cohlan’s studies detected over seventy types of birth defects that affected nearly every internal organ resulting from an excessive intake of vitamin A. Other scientists confirmed the effects of teratogenic and major birth defects due to an excess of vitamin A in different animal models, as well as the detrimental intake of high levels of vitamin A in the course of pregnancy [3,4,5,16]. For instance, the teratogenic effects of high RA in the kidney were demonstrated to be due to a local reduction of the RA level consequent to the downregulation of aldehyde dehydrogenases (ALDHs), which mediate RA synthesis, [17] as well as the upregulation of cytochrome P450 CYP26 enzymes, which induce the metabolic inactivation of RA [18]. These previously undescribed and unsuspected mechanisms provided insights into the molecular basis of RA-induced teratogenesis [19]. Apparently, the effects of vitamin A deficiency or toxicity are similar to symptoms induced by ethanol that, by affecting the physiological RA level [20], induces embryonic malformations [21].

The effects of RA and its dietary precursors in embryonic and fetal development are paralleled by the effects of RA and its dietary precursors in cancer. Chemoprevention trials in smokers, such as the Beta-Carotene and Retinol Efficacy Trial (CARET), the Alpha-Tocopherol, Beta-Carotene (ATBC) Cancer Prevention trial, and the Physicians’ Health Trial, showed that beta-carotene and retinol (vitamin A), rather than preventing cancer, induced detrimental tumorigenic effects in smokers [22,23,24,25]. Overall, these chemoprevention cancer trials revealed that different sources of RA, rather than reducing the incidence of cancer, promoted both cancer growth and invasion [26,27]. Similar studies performed by the National Cancer Institute Breast and Prostate Cancer Cohort Consortium and the European Prospective Investigation of Cancer (EPIC) on subjects with different cancer predisposing conditions (e.g., obesity, diabetes, defective immunity, and aging) did not lead to firm conclusions [28,29,30,31,32,33,34,35,36,37,38].

With the advent of cancer differentiation therapy, RA again took center stage [39]. RA, alone or in combination with arsenic trioxide (ATO), was shown to successfully treat acute promyelocytic leukemia (APL), which is an aggressive and fatal leukemia characterized by the differentiation arrest of promyelocytes, a very rapid downhill course, and severe bleeding caused by fibrinolysis. It is noteworthy that APL was the first cancer showing the dysregulation of ANXA8, which is a member of the Annexin family of calcium (Ca^2+^) and phospholipid binding proteins involved in several cellular functions [40]. The focus of this review is on emerging RA-regulated mechanisms involving specific annexins in APL and other cancers, with a special emphasis on cancers over-expressing ANXA8.

## 2. RA and Annexins in Myelopoiesis and Acute Promyelocytic Leukemia

RA is a potent morphogen of the hematopoietic development of human pluripotent stem cells. Genetic factors interfering with the normal physiological RA-RARA signaling pathways prevent cells from differentiating into mature promyelocytes [41,42,43,44,45,46,47]. More than forty years ago, the laboratory of Dr. Janet Rowley detected chromosome translocation in acute promyelocytic leukemia (APL) cells [48]. Remarkably, about 98% of APL patients show the translocation t (15; 17) (q24; q21) that generates an oncogenic fusion protein involving both RARA and a protein called PML, which is required for the assembly of PML-nuclear bodies surrounded by the chromatin of the nucleus. The PML nuclear bodies perform a number of regulatory cellular functions, including programmed cell death, genome stability, and cell division [49,50]. PML-RARA epigenetically repressed the chromatin associated with RARA target genes by recruiting corepressor complexes, including silencing mediator for retinoid and thyroid receptors (SMRT) and Nuclear Co-Repressor (NCOR), as well as histone deacetylases (HDACs) and DNA methyltransferases (DNMTs). Pharmacological RA, either alone or in combination with arsenic trioxide (ATO), relieved the epigenetic gene repression and restored promyelocytic differentiation by releasing corepressors and recruiting histone acetyl transferases (HATs) [51,52,53,54,55,56,57,58].

While the RA-induced differentiation of APL with PML-RARA was possible, other APLs did not respond to RA treatment. Specifically, a small percentage of APL patients were characterized by other RARA fusion proteins generated by different translocations. The t (11; 17) (q23; q21) that generates PLZF-RARA (currently named the ZBTB16-RARA protein) occurred in 1% of APL. The ZBTB16 transcriptional factor, which is involved in the self-renewal and differentiation of stem cells, recruited SMRT, NCOR, and HDACs that repressed RARA transcriptional target genes and blocked myeloid differentiation. The reciprocal protein RARA-ZBTB16 also contributed to leukemogenesis [59,60]. Notably, patients with ZBTB16-RARA were shown not to respond to either RA or arsenic trioxide (ATO). Even if RA degraded ZBTB16-RARA, it did not induce promyelocytic differentiation [61]. Other APL translocations were demonstrated to generate rare X-RARA fusion proteins, including NPM1-RARA [62], NUMA-RARA [63], and STAT5b-RARA [64]. The underlying molecular mechanisms of clinical resistance to RA and/or ATO are not all well-known in different X-RARA fusion proteins [65]. Interestingly, other rare variant APL fusion proteins do not involve RARA, but other RAR receptors, such as RA receptor beta (RARB) and RA receptor gamma (RARG) [66].

In classical APL, the PML-RARA fusion protein affected specific annexins, which are proteins capable of binding negatively charged phospholipids in membranes in a Ca^2+^-dependent manner [67] and are involved in major cellular processes (e.g., differentiation, proliferation, apoptosis, angiogenesis, vesiculation, membrane dynamics, cell migration, invasion, and adhesion) [34,68,69,70,71,72,73,74,75,76,77]. While PML-RARA induced a high expression of ANXA8 in APL [40,78,79], RA-induced downregulation of ANXA2 expression in myeloid leukemia cell lines was not confined to APL. Notably, high levels of ANXA2 in hyperfibrinolysis could be corrected by RA or RA plus ATO therapy in patients with APL [80,81,82].

Studies in mice showed that microRNAs (miRNAs) have a relevant role in hematopoiesis [83]. Human APL displayed a specific signature of miRNAs involved in regulatory loops of myeloid differentiation. For instance, the RA-regulated let-7a-3/let7-b cluster could distinguish APL blasts from normal promyelocytes [84]. One of the miRNAs upregulated by RA during APL differentiation was miR-342 [85]. This miRNA emerged as a direct transcriptional target of the critical hematopoietic transcription factor PU.1 and interferon regulatory factors IRF-1 and IRF-9. Apparently, IRF-1 maintains miR-342 at low levels, whereas the binding of PU.1 and IRF-9 in the promoter region, following RA-mediated differentiation, upregulated miR-342 expression. Moreover, the enforced expression of miR-342 in APL, promoted RA-induced differentiation [86]. Development and tumorigenesis are intimately connected. After the complex molecular networks and epigenetic mechanisms of myelopoiesis involving RA, RA receptors, annexins, and regulatory RNAs, the mammary gland represents another attractive developmental model.

## 3. RA and Annexins in Mammary Morphogenesis and Breast Cancer

### 3.1. Morphogenetic Effects of RA

Physiological RA is a major regulatory lipid signal and morphogen of mammary gland development. The rudimentary embryonic ductal tree remains quiescent until puberty and undergoes mammary gland branching morphogenesis under hormones’ stimulus. This is a unique model employed to study key morphogenetic and physiological processes. During a woman’s lifetime, the mammary gland also undergoes many changes in structure and function, including cyclic expansions corresponding to the hormonal changes induced by the menstrual cycle, as well as major changes during pregnancy, lactation, and involution. The dysregulation of signaling pathways of normal mammary gland development is due not only to intrinsic genetic factors, but also microenvironmental factors of the mammary gland extracellular matrix (ECM) and cell-cell interactions [87]. Physiological RA regulates all developmental stages of the mouse mammary gland by signaling via nuclear receptors with RA affinity.

**Figure 1:**
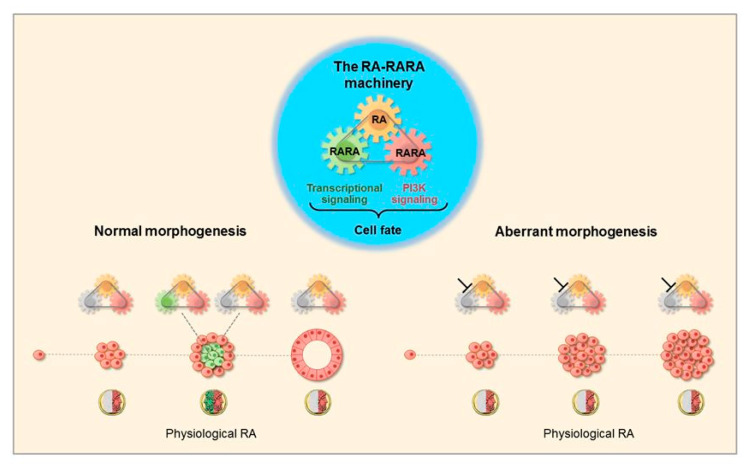
Physiological all-trans retinoic acid (RA) determines different cell fate decisions in normal and aberrant mammary epithelial cells. Blueprint of the three-module mechanism that integrates physiological RA and two RA receptor alpha (RARA) functions (center). In the course of normal human mammary epithelial cell morphogenesis (left scheme), physiological RA acts as the ancient two-faced Janus Bifrons god that regulates—in a spatiotemporal fashion—cell life (red) and cell death (green). Genetic factors affecting the RA-RARA mechanism induce 3D aberrant mammary morphogenesis (right scheme) [88].

Human normal mammary epithelial cells expressing wild type RARA seeded in basement membrane culture providing physiological RA formed 3D acinar structures with a lumen lined by apico-basal polarized cells. Factors affecting the functionality of the RA-RARA mechanism induced 3D amorphous structures with a lumen filled with proliferating cells (Figure 1) [88]. The inhibition of physiological RA signaling induced aberrant mammary morphogenesis in both mouse and human mammary epithelial cell models and in mouse mammary glands [89,90,91,92].

RARA was found to be expressed in several breast cancer cell lines (e.g., the RARA-positive T47D breast cancer cell line) [93]. In vitro pharmacological RA treatment of T47D cells was shown to induce either an anticancer or cancer-promoting action, depending on the functional status of RARA [94]. Consistently, in vivo mouse studies showed that T47D cells expressing wild type RARA and T47D cells with a dominant negative RARA mutant (DN-RARA^403^), when grown in nude mice fed a control diet providing physiological RA, developed dorsal xenograft tumors of similar sizes. However, T47D cells grown in nude mice fed an RA-enriched diet formed xenograft tumors smaller than xenograft tumors of mice fed a normal diet. In contrast, xenograft tumors of T47D cells carrying a dominant-negative mutant (DN-RARA^403^) fed the RA-enriched diet were significantly bigger than xenograft tumors of T47D with normal RARA [88].

The opposite effects of RA were induced in breast cancer cells via other nuclear receptors with affinity for RA. For instance, RA was transported by the Fatty Acid Binding Protein 5 (FABP5) on the Peroxisome Proliferator-Activated Receptor beta delta (PPARβ/δ) [95,96]. Alternatively, the opposite effects of RA on breast cancer cell growth were induced by either RARA or RAR gamma (RARG) [97,98,99].

### 3.2. The RA-RARA and ANXA8 Connection

Human isogenic mammary epithelial cell models, such as the MCF10A [100,101,102,103,104] and HME1 [88] models, revealed a connection between RA-RARA and ANXA8 [105,106,107,108,109,110]. The MCF10A cell line derived from a fibrocystic disease of a 36-year-old female was characterized by RARA expression, basal/myoepithelial, luminal markers, and stem/progenitor markers, including aldehyde dehydrogenase isoforms that regulate RA synthesis [111,112,113]. The MCF10A-1H cell line derived from premalignant MCF10A cells stably transfected with the human RAS oncogene formed aberrant 3D structures in vitro and tumors in mice. One of the mice tumors, which resembled a high grade subtype of human DCIS called comedocarcinoma, was later used to establish the MCF10A.com cell line (also MCF10A-DCIS.com) with features of comedocarcinoma in situ, representing a high grade subtype of human DCIS [100,101,102,103,104,114].

**Figure 2:**
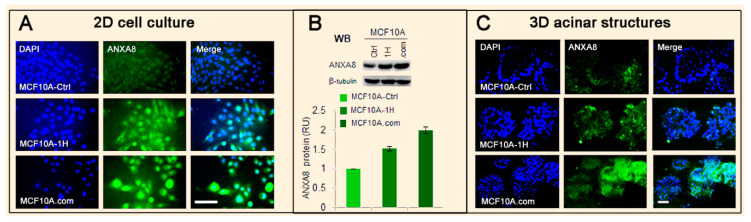
Evidence of increasing Annexin A8 (ANXA8) upregulation in isogenic MCF10A cell lines. (**A**) Immunofluorescence of MCF10A-Ctrl, MCF10A-1H, and MCF10A.com cell lines grown in two-dimensional (2D) culture stained with both 4′,6-diamidino-2-phenylindole (DAPI) (blue) and ANXA8 antibody (green) (scale bar 30 µm). (**B**) Western blotting (WB, top) and ANXA8 quantitative protein expression (bottom) in the three cell lines show an increasing ANXA8 level. (**C**) MCF10A-Ctrl, MCF10A-1H, and MCF10A.com cells grown in three-dimensional (3D) basement membrane culture (Matrigel) for 12 days were fixed and stained for DAPI and ANXA8 and imaged by confocal microscopy. MCF10A-Ctrl cells formed 3D acinar structures with a lumen lined by ANXA8-positive cells, while MCF10A-1H cells stably expressing the RAS oncogene developed morphologically aberrant acinar structures with ANXA8-positive cells in the luminal space. Comedocarcinoma MCF10A.com cells formed 3D ANXA8-positive “grape-like” morphologically aberrant structures (scale bar 30 µm).

The isogenic MCF10A-1H and MCF10.com cell lines with altered RA signaling [115] are characterized by incremental ANXA8 protein expression relative to the MCF10A control line (Figure 2).

**Figure 3:**
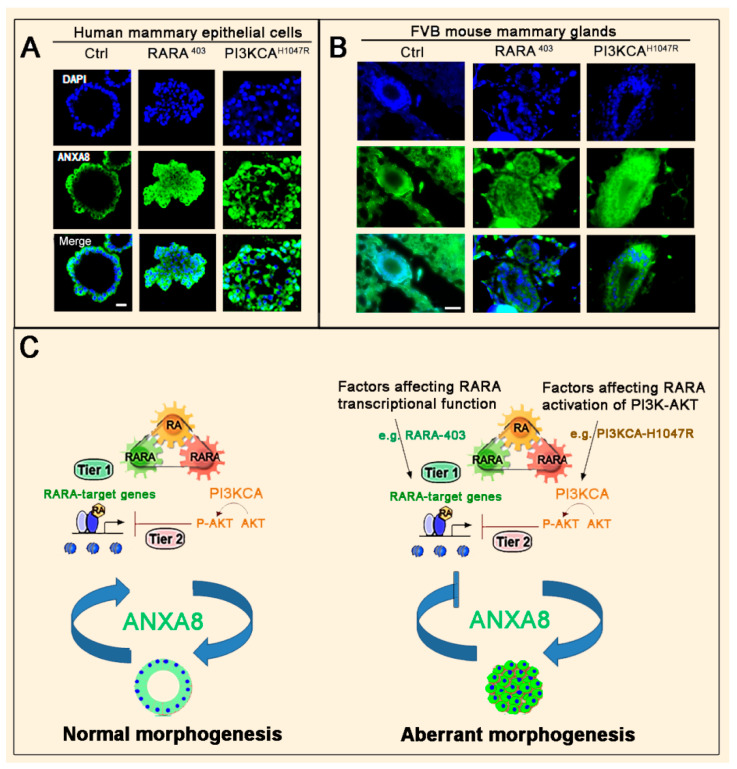
The RA-RARA-ANXA8 mechanism in normal and aberrant mammary morphogenesis. (**A**) Confocal microscopy images (scale bar: 10 µm) showing that normal human mammary epithelial (HME1) cells with normal RARA functions develop 3D acinar structures with a lumen lined by ANXA8-positive cells (left column). HME1 cells with RARA^403^ or PI3KCA^H1047R^ mutations develop ANXA8-positive, morphologically aberrant 3D acinar structures (middle and right columns) [110]. (**B**) Microscopy images of the fourth mammary gland section of a 12-week-old FVB female mouse with wild type RARA and wild type PI3KCA showing a duct lined by ANXA8-positive cells (left column). In contrast, the fourth mammary gland of 12-week-old FVB female mice expressing either MMTV-RARA^403^ or MMTV-PI3KCA^H1047R^ mutations showed ductal hyperplasia (DH) with ANXA8-positive cells in the luminal space (middle and right columns) (scale bar: 30 µm) (our unpublished images). (**C**) The interdependence of a functional RA-RARA mechanism and baseline ANXA8 expression generates normal morphogenesis (left scheme). In contrast, a defective RA-RARA mechanism upregulating endogenous ANXA8 generates aberrant morphogenesis (right scheme).

The human HME1 mammary epithelial cell line, which was created by the immortalization of primary human mammary epithelial cultures with telomerase, was demonstrated to exhibit monolayer growth in a cobblestone pattern characteristic of normal epithelial cells. HME1 cells with wild type RARA and wild type PI3KCA-AKT/PAKT functions developed 3D morphologically normal acinar structures with ANXA8-positive cells lining the luminal space in about 12 days (Figure 3A, left column). In contrast, HME1 cells with mutations (e.g., the dominant negative RARA^403^ or the mutant PI3KCA^H1047R^ p110 subunit) that affect the physiological RA transcriptional signaling developed 3D morphologically aberrant structures with a lumen filled with ANXA8-positive cells (Figure 3A, middle and right columns) [109,110].

Consistently, mammary glands of 12-week-old normal FVB female mice showed normal ducts lined by ANXA8-positive cells (Figure 3B, left column). In contrast, transgenic FVB female mice of the same age, expressing either MMTV-RARA^403^ or MMTV-PI3KCA^H1047R^ mutations, showed mammary glands with ductal hyperplasia (DH) characterized by an enlarged lumen filled with ANXA8-positive cells (Figure 3B, middle and right columns) similar to human breast DH [109].

As schematically shown in Figure 3C, the three-module RA-RARA mechanism of normal human and mouse mammary epithelial cells generated 3D acinar structures with a lumen lined by cells with baseline ANXA8 expression (left scheme). Endogenous ANXA8 overexpression induced by genetic factors generated aberrant morphogenesis driven by a downward spiral RA-RARA-ANXA8 mechanism that feeds back into itself (right scheme).

Elegant studies were conducted to monitor ANXA8 upregulation during mouse mammary gland development and involution [116], as well as in a large group of patients with breast cancer [117] and in subpopulations with transiently quiescent c-kit positive luminal cells of the ductal mammary epithelium [118]. Higher ANXA8 in ductal carcinoma in situ (DCIS) versus atypical ductal hyperplasia (ADH) and normal breast tissue was detected in tissue microarrays. Moreover, ANXA8 was found to be significantly overexpressed in Estrogen Receptor (ER)-negative versus ER-positive cases and significantly correlated with tumor stage, grade, and positive lymph nodes [109].

**Figure 4:**
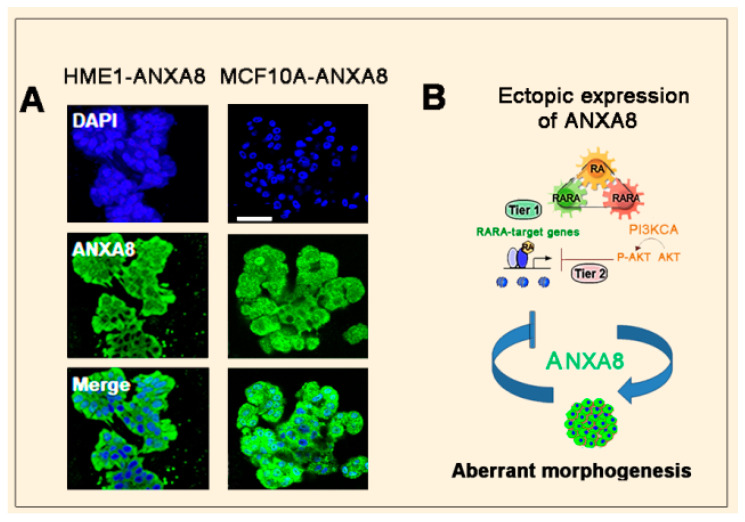
Ectopic ANXA8 generates aberrant mammary morphogenesis. (**A**) Confocal images of 3D morphologically aberrant structures generated by HME1 and MCF10A cells expressing stable ectopic ANXA8 (scale bar: 50 µm) (**B**) Scheme showing that ectopic ANXA8 is sufficient to induce aberrant morphogenesis.

Consistently, stable ectopic ANXA8 expression in HME1 and MCF10A cells induced 3D morphological aberrant structures (Figure 4A). Apparently, ectopic ANXA8, by repressing the transcription of RARA-target genes, generates a downward spiral mechanism of aberrant morphogenesis (Figure 4B) [108].

### 3.3. Mechanisms and Biomarkers Involving Regulatory microRNAs (miRNAs)

#### 3.3.1. Regulatory miRNAs of ANXA2 and ANXA8

Recent studies have suggested that RA, via RARA, can regulate the expression of ANXA8 and ANXA2 by direct transcriptional regulation and/or via microRNAs. Indeed, both the regulatory regions of ANXA8 and ANXA2 genes, and the regulatory regions of miRNAs targeting ANXA8 and ANXA2 mRNAs, are characterized by RA-responsive elements (RARE) [109].

**Figure 5:**
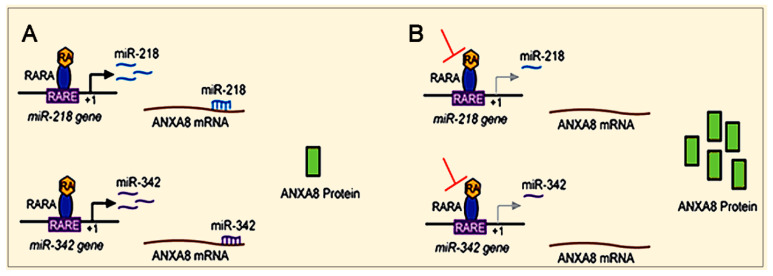
ANXA8 overexpression due to RA-RARA downregulation of ANXA8-regulatory miRNAs. (**A**) RA via RARA regulates ANXA8 regulatory miRNAs targeting the 3′untranslated region (3′UTR) of ANXA8 mRNA. (**B**) Factors inhibiting the RA-RARA regulation of ANXA8-regulatory miRNAs induce ANXA8 protein upregulation.

RA via RARA can induce baseline ANXA8 protein expression by regulating the transcription of specific miRNAs targeting the ANXA8 mRNA 3′untranslated region (3′UTR), including miR-218 associated with breast DCIS and miR-342 associated with triple negative breast cancer (TNBC) (Figure 5A) [109,119,120,121]. Factors (e.g., genetic mutations) that inhibit the RA-RARA transcriptional regulation of these miRNAs upregulate the ANXA8 protein (Figure 5B).

**Figure 6:**
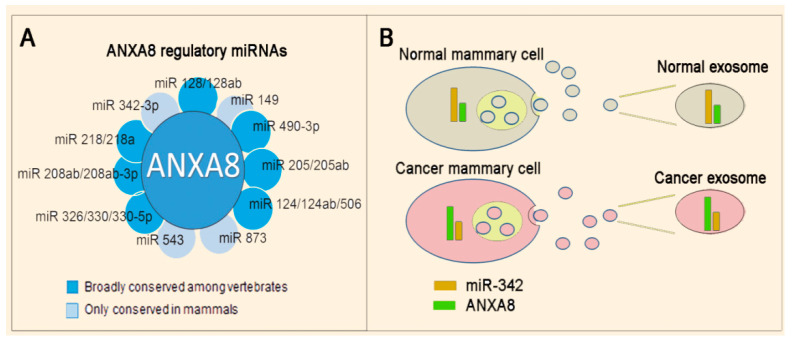
ANXA8-miRNA biomarkers. (**A**) Scheme showing a panel of eleven ANXA8 regulatory miRNAs detected by bioinformatics analysis [106]. (**B**) An inverse relationship of the ANXA8 protein and an ANXA8 regulatory miRNA (e.g., miR-342) in either normal or cancer mammary epithelial cells can also be detected in corresponding exosomes.

Bioinformatics analysis identified several ANXA8-regulatory miRNAs (Figure 6A) [110], including miRNAs (e.g., miR-342 shown in Figure 6B) that can be released in exosomes that are 50–140 nm vesicles containing proteins, mRNA, and miRNAs, shed by normal and cancer cells [122,123,124] and can be potentially detected in non-invasive liquid biopsies. By using methods that can selectively capture tumor-derived exosomes with antibodies against tumor-associated proteins and quantify in situ tumor-associated RNAs, it will be possible to discriminate between normal and malignant cells [125,126].

Several years ago, epigenetic markers such as hypermethylated RARB2 [105,106,127,128,129] enabled the detection of early stage breast cancer in ductal lavage fluid [130] and random periareolar fine needle aspirates [131]. Even if hypermethylated RARB2 has been proven useful for detecting RA-resistant breast cancer at pre-symptomatic stages, it has also shown some limitations (e.g., heterozygous RARB2 methylation may lead to false positives) [132]. Therefore, biomarkers such as RA-regulated miRNAs targeting ANXA8 would have several advantages over hypermethylated markers of RA-resistant breast cancer.

#### 3.3.2. Long Non-Coding RNAs (LncRNAs)

LncRNAs play important roles in breast cancer. They are involved in chromatin remodeling, as well as transcriptional and post-transcriptional regulation, through a variety of chromatin-based mechanisms and via cross-talk with other RNA species. For instance, lncRNA MALATI was shown to suppress breast cancer metastasis [133], while lncRNA DANCR was shown to upregulate PI3K/AKT signaling through the activation of the serine phosphorylation of RXRA [134,135].

There is scanty evidence of regulatory lncRNAs involved with annexins. However, lncRNA CCAT1, which is upregulated in breast cancer tissue and correlates with poor outcomes in breast cancer patients, was shown to interact with miR-204/211, miR-148a/152, and ANXA2. These interactions promoted the translocation of β-catenin to the nucleus, where it activated TCF4 that, in turn, activated the wingless/integrated (WNT) signaling. TCF4 binding of the promoter of lncCCAT1 regulated the lncCCAT1 transcription, and ultimately generated a positive feedback regulatory circuit of lncCCAT1-TCF4-lncCCAT1 in breast cancer stem cells [136]. Notably, a recent study showed that ANXA8 also regulates WNT signaling to maintain the phenotypic plasticity of retinal pigment epithelial cells [137].

## 4. Emerging Mechanisms Involving ANXA2 and/or ANXA8 in Different Cancers

According to a systematic literature search, different cancers are characterized by ANXA2 dysregulation [138]. Recently, several studies detecting ANXA8 upregulation in different cancers have been published. As shown hereafter, both ANXA2 and ANXA8 are not confined to acute promyelocytic leukemia and breast cancer, but are also involved in other cancers.

### 4.1. Prostate Cancer

Several published studies have suggested that the dysregulation of annexins plays a role in prostate cancer (PCa) progression through distinct mechanisms [139]. For instance, ANXA2 was found to be abundantly expressed in primary normal human prostate epithelial cells, but significantly reduced or lost in prostate cancer cell lines. ANXA2 was distributed in both the cytosol and underneath the plasma membrane in normal prostate epithelial cells, while prostate cancer cells showed reduced or lost expression in prostate cancer development and progression. ANXA2 re-expression inhibited prostate cancer cell migration [140,141]. In a recent large study, both ANXA8 and other markers were significantly overexpressed in metastatic castration-resistant prostate squamous cancer [142].

### 4.2. Ovarian Cancer

Most women diagnosed with ovarian cancer do not have a family history of ovarian cancer. However, women with a family member who has had ovarian and breast cancer have a higher risk. The most significant risk factors for ovarian cancer are inherited genetic mutations of BRCA1 or BRCA2 genes that are responsible for about 10 to 15 percent of all ovarian cancers. A recent study [143] showed that members of the Annexin family, including ANXA8, are involved in ovarian cancer tumorigenesis and progression. ANXA8 seemed to be involved in cell migration, cell adhesion, vasculature, and the regulation of PI3K-AKT. Immunocytochemistry showed that ANXA8 expression was higher in the malignant tumor group relative to borderline, benign, and normal groups. Furthermore, a high expression correlated with a poor prognosis. Based on a large study, ANXA8 might qualify as a novel biomarker for the early diagnosis, immunotherapy, and prognostic assessment of ovarian cancer. Notably, anticancer effects in ovarian serous cancer were induced by RA, which is not only a regulator of ANXA8, but also an inhibitor of the ANXA2-S100A10 signaling pathway [144].

### 4.3. Pancreatic Cancer

The worst prognosis of major cancers is for pancreatic cancer, since patients have a low survival rate [145]. ANXA8 expression was shown to be low or absent in normal pancreatic tissue, but was upregulated in pancreatic cancer [146,147]. Both Claudin 18 and ANXA8 have emerged as new markers of pancreatic cancer [148]. A pancreatic cancer study speculated that ANXA8 expression was low in a normal pancreas due to CpG hypermethylation of the ANXA8 promoter-exon 1 region, while demethylation of the ANXA8 promoter-exon 1 region induced ANXA8 upregulation in pancreatic cancer that invaded the surrounding tissue and metastasized [149]. Increased ANXA8 expression was associated with a poor prognosis [150]. Mechanistic studies showed that in pancreatic ductal adenocarcinoma (PDAC), ANXA8 upregulation was induced by the zinc finger regulator ZIC2, which is a member of the ZIC gene family that associates with GLI transcription factors of Hedgehog signaling [148,149,151,152]. A recent study showed that the long non-coding NHG14 RNA potentiates pancreatic cancer progression via ANXA2 modulation and acts as a competing endogenous RNA for miR-613 [153].

### 4.4. Gastric Carcinoma

Gastric carcinoma (GC) is the world’s leading cause of cancer-related death, especially among older males [154]. ANXA8 is notably elevated in GC tissues. A high expression of ANXA8 represents a poor prognosis of overall survival and disease-free survival, and significantly correlates with the TNM (T for the size of the tumor and any spread of cancer into nearby tissue, N for the spread of cancer to nearby lymph nodes, and M for metastasis) staging system that was created by the American Joint Committee on Cancer (AJCC) and the International Union Against Cancer (UICC). Further experimental validations are still required to clarify the molecular mechanisms underlying the role of ANXA8 in the course of GC progression [155].

### 4.5. Cholangiocarcinoma

Human cholangiocarcinoma (CC) arises from the biliary tree, which is a series of gastrointestinal ducts allowing newly synthesized bile from the liver to be concentrated and stored in the gallbladder prior to release into the duodenum. The common hepatic duct, which runs alongside the hepatic vein, descends and joins the cystic duct that combines and forms the common bile duct. Cholangiocarcinoma has a high mortality rate and a poor prognosis. The sarcomatous change/epithelial mesenchymal transition (EMT) of CC frequently leads to aggressive intrahepatic spread and metastasis. Two studies showed that ANXA8; S100P, which is a member of the S100 family of proteins containing two EF-hand calcium-binding motifs; and Glutathione Peroxidase 1 (GPX1) expression were decreased in CC dedifferentiation. ANXA8 was transcriptionally downregulated by the epidermal growth factor (EGF), which was correlated with morphological changes of the epithelial-mesenchymal transition (EMT). Ectopic ANXA8 reversed the morphology of cells in association with focal adhesion kinase expression and altered F-actin dynamics. The EGF receptor and its downstream targets PI3K kinase and AKT phosphorylated FOXO4 led to ANXA8 inhibition [156,157,158].

### 4.6. Oral Squamous Cell Carcinoma

The gene expression profiles of metastatic lymph nodes from oral squamous cell carcinoma (OSCC) patients relative to a control non-cancer cervical lymph node and heterotopic salivary gland tissue identified over eleven thousand genes, and found genes encoding ANXA8, keratin 6C (KRT6C), small proline-rich protein 1B (SPRR1B), and desmoglein 3 (DSG3) that were overexpressed in all metastatic lymph nodes. ANXA8 mRNA was detected in lymph nodes in which metastases were not identified histopathologically, but by the molecular RT-LAMP approach, which is more sensitive than conventional routine histopathology [158].

## 5. Conclusions

This review focuses on cancers expressing well-known annexins [138], with a special emphasis on ANXA8, which is an annexin that was found to be overexpressed for the first time in acute promyelocytic leukemia (APL) carrying a fusion gene involving both PML and RARA [40,78,79]. Subsequently, ANXA8 was extensively studied in the context of the mammary gland and breast cancer [116,117,118]. Consistently, ANXA8 upregulation was confirmed to be highly expressed in breast DCIS relative to ADH and normal breast tissue, as well as in 3D human mammary epithelial cell models of ductal carcinoma in situ (DCIS) [109,110].

More recently, ANXA8 was detected in several other cancers. After the original discovery of ANXA8 in classical APL several decades ago, research focusing on the role of ANXA8 in different cancers will be indispensable not only for developing diagnostic and prognostic biomarkers, but also for devising ad hoc therapeutic strategies. For instance, cancers expressing ANXA8 might be targeted with a strategy developed for targeting ANXA2 [159]. An increasing knowledge of miRNAs and lncRNAs with regulatory and epigenetic roles will be necessary to decipher tumorigenic mechanisms involving either single or multiple annexins.

## References

[1] Bushue N., Wan Y.J. (2010). Retinoid pathway and cancer therapeutics. Adv. Drug Deliv. Rev..

[2] Johnson E.J., Russell R.M., Coates P.M., Betz J.M., Blackman M.R., Cragg G.M., Levine M., Moss J., White J.D. (2010). Beta-carotene. Encyclopedia of Dietary Supplements.

[3] Ross A., Vitamin A., Shils M.S.M., Ross A., Caballero B., Cousins R. (2006). Carotenoids. Modern Nutrition in Health and Disease.

[4] Ross C.A., Vitamin A., Coates P.M., Betz J.M., Blackman M.R., Cragg G.M., Levine M., Moss J., White J.D. (2010). Encyclopedia of Dietary Supplements.

[5] Solomons N.W., Vitamin A., Bowman B., Russell R. (2006). Present Knowledge in Nutrition.

[6] Institute of Medicine (2001). Dietary Reference Intakes for Vitamin A, Vitamin K, Arsenic, Boron, Chromium, Copper, Iodine, Iron, Manganese, Molybdenum, Nickel, Silicon, Vanadium, and Zinc.

[7] Cabezuelo M.T., Zaragoza R., Barber T., Vina J.R. (2019). Role of vitamin a in mammary gland development and lactation. Nutrients.

[8] WHO (2009). Global Prevalence of Vitamin A Deficiency in Populations at Risk 1995–2005.

[9] Mayo-Wilson E., Imdad A., Herzer K., Yakoob M.Y., Bhutta Z.A. (2011). Vitamin A supplements for preventing mortality, illness, and blindness in children aged under 5: Systematic review and meta-analysis. BMJ.

[10] Sommer A. (2008). Vitamin a deficiency and clinical disease: An historical overview. J. Nutr..

[11] Darlow B.A., Graham P.J. (2016). Vitamin A supplementation to prevent mortality and short and long-term morbidity in very low birthweight infants. Cochrane Database Syst. Rev..

[12] Mactier H., Weaver L.T. (2005). Vitamin A and preterm infants: What we know, what we don’t know, and what we need to know. Arch. Dis. Child. Fetal Neonatal Ed..

[13] Oliveira-Menegozzo J.M., Bergamaschi D.P., Middleton P., East C.E. (2010). Vitamin A supplementation for postpartum women. Cochrane Database Syst. Rev..

[14] van den Broek N., Dou L., Othman M., Neilson J.P., Gates S., Gulmezoglu A.M. (2010). Vitamin A supplementation during pregnancy for maternal and newborn outcomes. Cochrane Database Syst. Rev..

[15] Cohlan S.Q. (1953). Excessive intake of vitamin A as a cause of congenital anomalies in the rat. Science.

[16] Ribaya-Mercado J.D., Blumberg J.B. (2007). Vitamin A: Is it a risk factor for osteoporosis and bone fracture?. Nutr. Rev..

[17] Niederreither K., Fraulob V., Garnier J.M., Chambon P., Dolle P. (2002). Differential expression of retinoic acid-synthesizing (RALDH) enzymes during fetal development and organ differentiation in the mouse. Mech. Dev..

[18] Ross A.C., Zolfaghari R. (2011). Cytochrome P450s in the regulation of cellular retinoic acid metabolism. Annu. Rev. Nutr..

[19] Lee L.M., Leung C.Y., Tang W.W., Choi H.L., Leung Y.C., McCaffery P.J., Wang C.C., Woolf A.S., Shum A.S. (2012). A paradoxical teratogenic mechanism for retinoic acid. Proc. Natl. Acad. Sci. USA.

[20] Napoli J.L. (2011). Effects of ethanol on physiological retinoic acid levels. IUBMB Life.

[21] Kot-Leibovich H., Fainsod A. (2009). Ethanol induces embryonic malformations by competing for retinaldehyde dehydrogenase activity during vertebrate gastrulation. Dis. Model Mech..

[22] Freemantle S.J., Dragnev K.H., Dmitrovsky E. (2006). The retinoic acid paradox in cancer chemoprevention. J. Natl. Cancer Inst..

[23] Omenn G.S., Goodman G.E., Thornquist M.D., Balmes J., Cullen M.R., Glass A., Keogh J.P., Meyskens F.L., Valanis B., Williams J.H. (1996). Effects of a combination of beta carotene and vitamin A on lung cancer and cardiovascular disease. N. Engl. J. Med..

[24] Hennekens C.H., Buring J.E., Manson J.E., Stampfer M., Rosner B., Cook N.R., Belanger C., LaMotte F., Gaziano J.M., Ridker P.M. (1996). Lack of effect of long-term supplementation with beta carotene on the incidence of malignant neoplasms and cardiovascular disease. N. Engl. J. Med..

[25] The Alpha-Tocopherol Beta-Carotene Cancer Prevention Study Group (1994). The effect of vitamin E and beta carotene on the incidence of lung cancer and other cancers in male smokers. N. Engl. J. Med..

[26] Khuri F.R., Lee J.J., Lippman S.M., Kim E.S., Cooper J.S., Benner S.E., Winn R., Pajak T.F., Williams B., Shenouda G. (2006). Randomized phase III trial of low-dose isotretinoin for prevention of second primary tumors in stage I and II head and neck cancer patients. J. Natl. Cancer Inst..

[27] Lippman S.M., Lee J.J., Karp D.D., Vokes E.E., Benner S.E., Goodman G.E., Khuri F.R., Marks R., Winn R.J., Fry W. (2001). Randomized phase III intergroup trial of isotretinoin to prevent second primary tumors in stage I non-small-cell lung cancer. J. Natl. Cancer Inst..

[28] Blaner W.S. (2019). Vitamin A signaling and homeostasis in obesity, diabetes, and metabolic disorders. Pharmacol. Ther..

[29] Ecker B.L., Lee J.Y., Sterner C.J., Solomon A.C., Pant D.K., Shen F., Peraza J., Vaught L., Mahendra S., Belka G.K. (2019). Impact of obesity on breast cancer recurrence and minimal residual disease. Breast Cancer Res..

[30] Ross A.C. (2012). Vitamin A and retinoic acid in T cell-related immunity. Am. J. Clin. Nutr..

[31] Brown C.C., Esterhazy D., Sarde A., London M., Pullabhatla V., Osma-Garcia I., Al-Bader R., Ortiz C., Elgueta R., Arno M. (2015). Retinoic acid is essential for Th1 cell lineage stability and prevents transition to a Th17 cell program. Immunity.

[32] LaBarge M.A., Mora-Blanco E.L., Samson S., Miyano M. (2016). Breast cancer beyond the age of mutation. Gerontology.

[33] Todhunter M.E., LaBarge M.A. (2017). Cell and tissue biology paves a path to breast cancer prevention. Trends Cancer.

[34] Hendrickson S.J., Lindstrom S., Eliassen A.H., Rosner B.A., Chen C., Barrdahl M., Brinton L., Buring J., Canzian F., Chanock S. (2013). Plasma carotenoid- and retinol-weighted multi-SNP scores and risk of breast cancer in the National Cancer Institute Breast and Prostate Cancer Cohort Consortium. Cancer Epidemiol. Biomark. Prev..

[35] Eliassen A.H., Hendrickson S.J., Brinton L.A., Buring J.E., Campos H., Dai Q., Dorgan J.F., Franke A.A., Gao Y.T., Goodman M.T. (2012). Circulating carotenoids and risk of breast cancer: Pooled analysis of eight prospective studies. J. Natl. Cancer Inst..

[36] Eliassen A.H., Liao X., Rosner B., Tamimi R.M., Tworoger S.S., Hankinson S.E. (2015). Plasma carotenoids and risk of breast cancer over 20 y of follow-up. Am. J. Clin. Nutr..

[37] Nagel G., Linseisen J., van Gils C.H., Peeters P.H., Boutron-Ruault M.C., Clavel-Chapelon F., Romieu I., Tjonneland A., Olsen A., Roswall N. (2010). Dietary beta-carotene, vitamin C and E intake and breast cancer risk in the European Prospective Investigation into Cancer and Nutrition (EPIC). Breast Cancer Res. Treat.

[38] Bakker M.F., Peeters P.H., Klaasen V.M., Bueno-de-Mesquita H.B., Jansen E.H., Ros M.M., Travier N., Olsen A., Tjonneland A., Overvad K. (2016). Plasma carotenoids, vitamin C, tocopherols, and retinol and the risk of breast cancer in the European Prospective Investigation into Cancer and Nutrition cohort. Am. J. Clin. Nutr..

[39] Sachs L. (1978). The differentiation of myeloid leukaemia cells: New possibilities for therapy. Br. J. Haematol..

[40] Chang K.S., Wang G., Freireich E.J., Daly M., Naylor S.L., Trujillo J.M., Stass S.A. (1992). Specific expression of the annexin VIII gene in acute promyelocytic leukemia. Blood.

[41] Stavem P. (1978). Acute hypergranular promyelocytic leukaemia. Priority of discovery (Jean Bernard and Leif Hillestad). Scand J. Haematol..

[42] Collins S.J., Gallo R.C., Gallagher R.E. (1977). Continuous growth and differentiation of human myeloid leukaemic cells in suspension culture. Nature.

[43] Breitman T.R., Selonick S.E., Collins S.J. (1980). Induction of differentiation of the human promyelocytic leukemia cell line (HL-60) by retinoic acid. Proc. Natl. Acad. Sci. USA.

[44] Koeffler H.P. (1983). Induction of differentiation of human acute myelogenous leukemia cells: Therapeutic implications. Blood.

[45] Chomienne C., Ballerini P., Balitrand N., Amar M., Bernard J.F., Boivin P., Daniel M.T., Berger R., Castaigne S., Degos L. (1989). Retinoic acid therapy for promyelocytic leukaemia. Lancet.

[46] Chen Z.X., Xue Y.Q., Zhang R., Tao R.F., Xia X.M., Li C., Wang W., Zu W.Y., Yao X.Z., Ling B.J. (1991). A clinical and experimental study on all-trans retinoic acid-treated acute promyelocytic leukemia patients. Blood.

[47] Rao Y., Li R., Zhang D. (2013). A drug from poison: How the therapeutic effect of arsenic trioxide on acute promyelocytic leukemia was discovered. Sci. China Life Sci..

[48] Rowley J.D., Golomb H.M., Dougherty C. (1977). 15/17 translocation, a consistent chromosomal change in acute promyelocytic leukaemia. Lancet.

[49] Bernardi R., Pandolfi P.P. (2007). Structure, dynamics and functions of promyelocytic leukaemia nuclear bodies. Nat. Rev. Mol. Cell Biol..

[50] Sahin U., Lallemand-Breitenbach V., de The H. (2014). PML nuclear bodies: Regulation, function and therapeutic perspectives. J. Pathol..

[51] Huang M.E., Ye Y.C., Chen S.R., Chai J.R., Lu J.X., Zhoa L., Gu L.J., Wang Z.Y. (1988). Use of all-trans retinoic acid in the treatment of acute promyelocytic leukemia. Blood.

[52] Kakizuka A., Miller W.H., Umesono K., Warrell R.P., Frankel S.R., Murty V.V., Dmitrovsky E., Evans R.M. (1991). Chromosomal translocation t(15;17) in human acute promyelocytic leukemia fuses RAR alpha with a novel putative transcription factor, PML. Cell.

[53] Wang Z.Y., Chen Z. (2008). Acute promyelocytic leukemia: From highly fatal to highly curable. Blood.

[54] Carbone R., Botrugno O.A., Ronzoni S., Insinga A., di Croce L., Pelicci P.G., Minucci S. (2006). Recruitment of the histone methyltransferase SUV39H1 and its role in the oncogenic properties of the leukemia-associated PML-retinoic acid receptor fusion protein. Mol. Cell Biol..

[55] Villa R., Pasini D., Gutierrez A., Morey L., Occhionorelli M., Vire E., Nomdedeu J.F., Jenuwein T., Pelicci P.G., Minucci S. (2007). Role of the polycomb repressive complex 2 in acute promyelocytic leukemia. Cancer Cell.

[56] Grignani F., de Matteis S., Nervi C., Tomassoni L., Gelmetti V., Cioce M., Fanelli M., Ruthardt M., Ferrara F.F., Zamir I. (1998). Fusion proteins of the retinoic acid receptor-alpha recruit histone deacetylase in promyelocytic leukaemia. Nature.

[57] Nowak D., Stewart D., Koeffler H.P. (2009). Differentiation therapy of leukemia: 3 decades of development. Blood.

[58] de The H., Pandolfi P.P., Chen Z. (2017). Acute Promyelocytic leukemia: A Paradigm for oncoprotein-targeted cure. Cancer Cell.

[59] He L.Z., Bhaumik M., Tribioli C., Rego E.M., Ivins S., Zelent A., Pandolfi P.P. (2000). Two critical hits for promyelocytic leukemia. Mol. Cell.

[60] Guidez F., Parks S., Wong H., Jovanovic J.V., Mays A., Gilkes A.F., Mills K.I., Guillemin M.C., Hobbs R.M., Pandolfi P.P. (2007). RARalpha-PLZF overcomes PLZF-mediated repression of CRABPI, contributing to retinoid resistance in t(11;17) acute promyelocytic leukemia. Proc. Natl. Acad. Sci. USA.

[61] Lehmann-Che J., Bally C., de The H. (2014). Resistance to therapy in acute promyelocytic leukemia. N. Engl. J. Med..

[62] Sainty D., Liso V., Cantu-Rajnoldi A., Head D., Mozziconacci M.J., Arnoulet C., Benattar L., Fenu S., Mancini M., Duchayne E. (2000). A new morphologic classification system for acute promyelocytic leukemia distinguishes cases with underlying PLZF/RARA gene rearrangements. Blood.

[63] Wells R.A., Catzavelos C., Kamel-Reid S. (1997). Fusion of retinoic acid receptor alpha to NuMA, the nuclear mitotic apparatus protein, by a variant translocation in acute promyelocytic leukaemia. Nat. Genet..

[64] Arnould C., Philippe C., Bourdon V., goire M.J.G., Berger R., Jonveaux P. (1999). The signal transducer and activator of transcription STAT5b gene is a new partner of retinoic acid receptor alpha in acute promyelocytic-like leukaemia. Hum Mol. Genet..

[65] Noguera N.I., Catalano G., Banella C., Divona M., Faraoni I., Ottone T., Arcese W., Voso M.T. (2019). Acute promyelocytic leukemia: Update on the mechanisms of leukemogenesis, resistance and on innovative treatment strategies. Cancers.

[66] Geoffroy M.C., de The H. (2020). Classic and variants APLs, as viewed from a therapy response. Cancers.

[67] Monastyrskaya K. (2018). Functional association between regulatory RNAs and the annexins. Int. J. Mol. Sci..

[68] Gerke V., Moss S.E. (2002). Annexins: From structure to function. Physiol. Rev..

[69] Gerke V., Creutz C.E., Moss S.E. (2005). Annexins: Linking Ca2+ signalling to membrane dynamics. Nat. Rev. Mol. Cell Biol..

[70] Raynal P., Pollard H.B. (1994). Annexins: The problem of assessing the biological role for a gene family of multifunctional calcium- and phospholipid-binding proteins. Biochim. Biophys. Acta.

[71] Bandorowicz J., Pikula S. (1993). Annexins--multifunctional, calcium-dependent, phospholipid-binding proteins. Acta Biochim. Pol..

[72] Satoh A., Miwa H.E., Kojima K., Hirabayashi J., Matsumoto I. (2000). Ligand-binding properties of annexin from Caenorhabditis elegans (annexin XVI, Nex-1). J. Biochem..

[73] Rescher U., Gerke V. (2004). Annexins—Unique membrane binding proteins with diverse functions. J. Cell Sci..

[74] Rosengarth A., Luecke H. (2003). A calcium-driven conformational switch of the N-terminal and core domains of annexin A1. J. Mol. Biol..

[75] Faure A.V., Migne C., Devilliers G., Ayala-Sanmartin J. (2002). Annexin 2 “secretion” accompanying exocytosis of chromaffin cells: Possible mechanisms of annexin release. Exp. Cell Res..

[76] Benaud C., Gentil B.J., Assard N., Court M., Garin J., Delphin C., Baudier J. (2004). AHNAK interaction with the annexin 2/S100A10 complex regulates cell membrane cytoarchitecture. J. Cell Biol..

[77] Jacob R., Heine M., Eikemeyer J., Frerker N., Zimmer K.P., Rescher U., Gerke V., Naim H.Y. (2004). Annexin II is required for apical transport in polarized epithelial cells. J. Biol. Chem..

[78] Sarkar A., Yang P., Fan Y.H., Mu Z.M., Hauptmann R., Adolf G.R., Stass S.A., Chang K.S. (1994). Regulation of the expression of annexin VIII in acute promyelocytic leukemia. Blood.

[79] Liu J.H., Stass S.A., Chang K.S. (1994). Expression of the annexin VIII gene in acute promyelocytic leukemia. Leuk. Lymphoma.

[80] Olwill S.A., McGlynn H., Gilmore W.S., Alexander H.D. (2005). All-trans retinoic acid-induced downregulation of annexin II expression in myeloid leukaemia cell lines is not confined to acute promyelocytic leukaemia. Br. J. Haematol..

[81] Kwaan H.C., Weiss I., Tallman M.S. (2019). The role of abnormal hemostasis and fibrinolysis in morbidity and mortality of acute promyelocytic leukemia. Semin. Thromb. Hemost..

[82] Huang D., Yang Y., Sun J., Dong X., Wang J., Liu H., Lu C., Chen X., Shao J., Yan J. (2017). Annexin A2-S100A10 heterotetramer is upregulated by PML/RARalpha fusion protein and promotes plasminogen-dependent fibrinolysis and matrix invasion in acute promyelocytic leukemia. Front. Med..

[83] Chen C.Z., Li L., Lodish H.F., Bartel D.P. (2004). MicroRNAs modulate hematopoietic lineage differentiation. Science.

[84] Garzon R., Pichiorri F., Palumbo T., Visentini M., Aqeilan R., Cimmino A., Wang H., Sun H., Volinia S., Alder H. (2007). MicroRNA gene expression during retinoic acid-induced differentiation of human acute promyelocytic leukemia. Oncogene.

[85] Careccia S., Mainardi S., Pelosi A., Gurtner A., Diverio D., Riccioni R., Testa U., Pelosi E., Piaggio G., Sacchi A. (2009). A restricted signature of miRNAs distinguishes APL blasts from normal promyelocytes. Oncogene.

[86] de Marchis M.L., Ballarino M., Salvatori B., Puzzolo M.C., Bozzoni I., Fatica A. (2009). A new molecular network comprising PU.1, interferon regulatory factor proteins and miR-342 stimulates ATRA-mediated granulocytic differentiation of acute promyelocytic leukemia cells. Leukemia.

[87] Inman J.L., Robertson C., Mott J.D., Bissell M.J. (2015). Mammary gland development: Cell fate specification, stem cells and the microenvironment. Development.

[88] Rossetti S., Ren M., Visconti N., Corlazzoli F., Gagliostro V., Somenzi G., Yao J., Sun Y., Sacchi N. (2016). Tracing anti-cancer and cancer-promoting actions of all-trans retinoic acid in breast cancer to a RARalpha epigenetic mechanism of mammary epithelial cell fate. Oncotarget.

[89] Montesano R., Soulie P. (2002). Retinoids induce lumen morphogenesis in mammary epithelial cells. J. Cell Sci..

[90] Wang Y.A., Shen K., Wang Y., Brooks S.C. (2005). Retinoic acid signaling is required for proper morphogenesis of mammary gland. Dev. Dyn..

[91] Cho K.W., Kwon H.J., Shin J.O., Lee J.M., Cho S.W., Tickle C., Jung H.S. (2012). Retinoic acid signaling and the initiation of mammary gland development. Dev. Biol..

[92] Cohn E., Ossowski L., Bertran S., Marzan C., Farias E.F. (2010). RARalpha1 control of mammary gland ductal morphogenesis and wnt1-tumorigenesis. Breast Cancer Res..

[93] Centritto F., Paroni G., Bolis M., Garattini S.K., Kurosaki M., Barzago M.M., Zanetti A., Fisher J.N., Scott M.F., Pattini L. (2015). Cellular and molecular determinants of all-trans retinoic acid sensitivity in breast cancer: Luminal phenotype and RARalpha expression. EMBO Mol. Med..

[94] Somenzi G., Sala G., Rossetti S., Ren M., Ghidoni R., Sacchi N. (2007). Disruption of retinoic acid receptor alpha reveals the growth promoter face of retinoic acid. PLoS ONE.

[95] Schug T.T., Berry D.C., Shaw N.S., Travis S.N., Noy N. (2007). Opposing effects of retinoic acid on cell growth result from alternate activation of two different nuclear receptors. Cell.

[96] Schug T.T., Berry D.C., Toshkov I.A., Cheng L., Nikitin A.Y., Noy N. (2008). Overcoming retinoic acid-resistance of mammary carcinomas by diverting retinoic acid from PPARbeta/delta to RAR. Proc. Natl. Acad. Sci. USA.

[97] Bosch A., Bertran S.P., Lu Y., Garcia A., Jones A.M., Dawson M.I., Farias E.F. (2012). Reversal by RARalpha agonist Am580 of c-Myc-induced imbalance in RARalpha/RARgamma expression during MMTV-Myc tumorigenesis. Breast Cancer Res..

[98] Garattini E., Bolis M., Garattini S.K., Fratelli M., Centritto F., Paroni G., Gianni M., Zanetti A., Pagani A., Fisher J.N. (2014). Retinoids and breast cancer: From basic studies to the clinic and back again. Cancer Treat. Rev..

[99] Garattini E., Paroni G., Terao M. (2012). Retinoids and breast cancer: New clues to increase their activity and selectivity. Breast Cancer Res..

[100] Dawson P.J., Wolman S.R., Tait L., Heppner G.H., Miller F.R. (1996). MCF10AT: A model for the evolution of cancer from proliferative breast disease. Am. J. Pathol..

[101] Miller F.R., Santner S.J., Tait L., Dawson P.J. (2000). MCF10DCIS.com xenograft model of human comedo ductal carcinoma in situ. J. Natl. Cancer Inst..

[102] Miller F.R., Soule H.D., Tait L., Pauley R.J., Wolman S.R., Dawson P.J., Heppner G.H. (1993). Xenograft model of progressive human proliferative breast disease. J. Natl. Cancer Inst..

[103] Behbod F., Kittrell F.S., LaMarca H., Edwards D., Kerbawy S., Heestand J.C., Young E., Mukhopadhyay P., Yeh H.W., Allred D.C. (2009). An intraductal human-in-mouse transplantation model mimics the subtypes of ductal carcinoma in situ. Breast Cancer Res..

[104] Imbalzano K.M., Tatarkova I., Imbalzano A.N., Nickerson J.A. (2009). Increasingly transformed MCF-10A cells have a progressively tumor-like phenotype in three-dimensional basement membrane culture. Cancer Cell Int..

[105] Bistulfi G., Pozzi S., Ren M., Rossetti S., Sacchi N. (2006). A repressive epigenetic domino effect confers susceptibility to breast epithelial cell transformation: Implications for predicting breast cancer risk. Cancer Res..

[106] Corlazzoli F., Rossetti S., Bistulfi G., Ren M., Sacchi N. (2009). Derangement of a factor upstream of RARalpha triggers the repression of a pleiotropic epigenetic network. PLoS ONE.

[107] Rossetti S., Hoogeveen A.T., Esposito J., Sacchi N. (2010). Loss of MTG16a (CBFA2T3), a novel rDNA repressor, leads to increased ribogenesis and disruption of breast acinar morphogenesis. J. Cell Mol. Med..

[108] Rossetti S., Corlazzoli F., Gregorski A., Azmi N.H., Sacchi N. (2012). Identification of an estrogen-regulated circadian mechanism necessary for breast acinar morphogenesis. Cell Cycle.

[109] Rossetti S., Bshara W., Reiners J.A., Corlazzoli F., Miller A., Sacchi N. (2016). Harnessing 3D models of mammary epithelial morphogenesis: An off the beaten path approach to identify candidate biomarkers of early stage breast cancer. Cancer Lett..

[110] Rossetti S., Sacchi N. (2019). 3D mammary epithelial cell models: A goldmine of DCIS Biomarkers and morphogenetic mechanisms. Cancers.

[111] Ginestier C., Hur M.H., Charafe-Jauffret E., Monville F., Dutcher J., Brown M., Jacquemier J., Viens P., Kleer C.G., Liu S. (2007). ALDH1 is a marker of normal and malignant human mammary stem cells and a predictor of poor clinical outcome. Cell Stem Cell.

[112] Ma I., Allan A.L. (2011). The role of human aldehyde dehydrogenase in normal and cancer stem cells. Stem Cell Rev. Rep..

[113] Liu S., Cong Y., Wang D., Sun Y., Deng L., Liu Y., Martin-Trevino R., Shang L., McDermott S.P., Landis M.D. (2014). Breast cancer stem cells transition between epithelial and mesenchymal states reflective of their normal counterparts. Stem Cell Rep..

[114] Badve S.S., Gokmen-Polar Y. (2019). Ductal carcinoma in situ of breast: Update 2019. Pathology.

[115] Peng X., Yun D., Christov K. (2004). Breast cancer progression in MCF10A series of cell lines is associated with alterations in retinoic acid and retinoid X receptors and with differential response to retinoids. Int. J. Oncol..

[116] Stein T., Morris J.S., Davies C.R., Weber-Hall S.J., Duffy M.A., Heath V.J., Bell A.K., Ferrier R.K., Sandilands G.P., Gusterson B.A. (2004). Involution of the mouse mammary gland is associated with an immune cascade and an acute-phase response, involving LBP, CD14 and STAT3. Breast Cancer Res..

[117] Stein T., Price K.N., Morris J.S., Heath V.J., Ferrier R.K., Bell A.K., Pringle M.A., Villadsen R., Petersen O.W., Sauter G. (2005). Annexin A8 is up-regulated during mouse mammary gland involution and predicts poor survival in breast cancer. Clin. Cancer Res..

[118] Iglesias J.M., Cairney C.J., Ferrier R.K., McDonald L., Soady K., Kendrick H., Pringle M.A., Morgan R.O., Martin F., Smalley M.J. (2015). Annexin A8 identifies a subpopulation of transiently quiescent c-kit positive luminal progenitor cells of the ductal mammary epithelium. PLoS ONE.

[119] Sanchez-Gonzalez I., Bobien A., Molnar C., Schmid S., Strotbek M., Boerries M., Busch H., Olayioye M.A. (2020). miR-149 suppresses breast cancer metastasis by blocking paracrine interactions with macrophages. Cancer Res..

[120] Romero-Cordoba S.L., Rodriguez-Cuevas S., Bautista-Pina V., Maffuz-Aziz A., D’Ippolito E., Cosentino G., Baroni S., Iorio M.V., Hidalgo-Miranda A. (2018). Loss of function of miR-342-3p results in MCT1 over-expression and contributes to oncogenic metabolic reprogramming in triple negative breast cancer. Sci. Rep..

[121] Young J., Kawaguchi T., Yan L., Qi Q., Liu S., Takabe K. (2017). Tamoxifen sensitivity-related microRNA-342 is a useful biomarker for breast cancer survival. Oncotarget.

[122] Halvaei S., Daryani S., Eslami S.Z., Samadi T., Jafarbeik-Iravani N., Bakhshayesh T.O., Majidzadeh A.K., Esmaeili R. (2018). Exosomes in cancer liquid biopsy: A focus on breast cancer. Mol. Ther. Nucleic Acids.

[123] Alimirzaie S., Bagherzadeh M., Akbari M.R. (2019). Liquid biopsy in breast cancer: A comprehensive review. Clin. Genet..

[124] Lennon A.M., Buchanan A.H., Kinde I., Warren A., Honushefsky A., Cohain A.T., Ledbetter D.H., Sanfilippo F., Sheridan K., Rosica D. (2020). Feasibility of blood testing combined with PET-CT to screen for cancer and guide intervention. Science.

[125] Chen S.L., Chen C.Y., Hsieh J.C., Yu Z.Y., Cheng S.J., Hsieh K.Y., Yang J.W., Kumar P.V., Lin S.F., Chen G.Y. (2019). Graphene oxide-based biosensors for liquid biopsies in cancer diagnosis. Nanomaterials.

[126] Yang Y., Kannisto E., Yu G., Reid M.E., Patnaik S.K., Wu Y. (2018). An immuno-biochip selectively captures tumor-derived exosomes and detects exosomal RNAs for cancer diagnosis. ACS Appl. Mater. Interfaces.

[127] Ren M., Pozzi S., Bistulfi G., Somenzi G., Rossetti S., Sacchi N. (2005). Impaired retinoic acid (RA) signal leads to RARbeta2 epigenetic silencing and RA resistance. Mol. Cell Biol..

[128] Sirchia S.M., Ferguson A.T., Sironi E., Subramanyan S., Orlandi R., Sukumar S., Sacchi N. (2000). Evidence of epigenetic changes affecting the chromatin state of the retinoic acid receptor beta2 promoter in breast cancer cells. Oncogene.

[129] Sirchia S.M., Ren M., Pili R., Sironi E., Somenzi G., Ghidoni R., Toma S., Nicolo G., Sacchi N. (2002). Endogenous reactivation of the RARbeta2 tumor suppressor gene epigenetically silenced in breast cancer. Cancer Res..

[130] Evron E., Dooley W.C., Umbricht C.B., Rosenthal D., Sacchi N., Gabrielson E., Soito A.B., Hung D.T., Ljung B., Davidson N.E. (2001). Detection of breast cancer cells in ductal lavage fluid by methylation-specific PCR. Lancet.

[131] Bean G.R., Scott V., Yee L., Ratliff-Daniel B., Troch M.M., Seo P., Bowie M.L., Marcom P.K., Slade J., Kimler B.F. (2005). Retinoic acid receptor-beta2 promoter methylation in random periareolar fine needle aspiration. Cancer Epidemiol Biomark. Prev..

[132] Fang C., Jian Z.Y., Shen X.F., Wei X.M., Yu G.Z., Zeng X.T. (2015). Promoter methylation of the retinoic acid receptor beta2 (RARbeta2) Is associated with increased risk of breast cancer: A PRISMA compliant meta-analysis. PLoS ONE.

[133] Kim J., Piao H.L., Kim B.J., Yao F., Han Z., Wang Y., Xiao Z., Siverly A.N., Lawhon S.E., Ton B.N. (2018). Long noncoding RNA MALAT1 suppresses breast cancer metastasis. Nat. Genet..

[134] Tang J., Zhong G., Zhang H., Yu B., Wei F., Luo L., Kang Y., Wu J., Jiang J., Li Y. (2018). LncRNA DANCR upregulates PI3K/AKT signaling through activating serine phosphorylation of RXRA. Cell Death Dis..

[135] Zhang T., Hu H., Yan G., Wu T., Liu S., Chen W., Ning Y., Lu Z. (2019). Long non-coding RNA and breast cancer. Technol. Cancer Res. Treat..

[136] Tang T., Guo C., Xia T., Zhang R., Zen K., Pan Y., Jin L. (2019). LncCCAT1 promotes breast cancer stem cell function through activating WNT/beta-catenin signaling. Theranostics.

[137] Lueck K., Carr A.F., Yu L., Greenwood J., Moss S.E. (2020). Annexin A8 regulates wnt signaling to maintain the phenotypic plasticity of retinal pigment epithelial cells. Sci. Rep..

[138] Christensen M.V., Hogdall C.K., Jochumsen K.M., Hogdall E.V.S. (2018). Annexin A2 and cancer: A systematic review. Int. J. Oncol..

[139] Xin W., Rhodes D.R., Ingold C., Chinnaiyan A.M., Rubin M.A. (2003). Dysregulation of the annexin family protein family is associated with prostate cancer progression. Am. J. Pathol..

[140] Liu J.W., Shen J.J., Tanzillo-Swarts A., Bhatia B., Maldonado C.M., Person M.D., Lau S.S., Tang D.G. (2003). Annexin II expression is reduced or lost in prostate cancer cells and its re-expression inhibits prostate cancer cell migration. Oncogene.

[141] Ardura J.A., Alvarez-Carrion L., Gutierrez-Rojas I., Alonso V. (2020). Role of calcium signaling in prostate cancer progression: Effects on cancer hallmarks and bone metastatic mechanisms. Cancers.

[142] Labrecque M.P., Coleman I.M., Brown L.G., True L.D., Kollath L., Lakely B., Nguyen H.M., Yang Y.C., da Costa R.M.G., Kaipainen A. (2019). Molecular profiling stratifies diverse phenotypes of treatment-refractory metastatic castration-resistant prostate cancer. J. Clin. Investig..

[143] Gou R., Zhu L., Zheng M., Guo Q., Hu Y., Li X., Liu J., Lin B. (2019). Annexin A8 can serve as potential prognostic biomarker and therapeutic target for ovarian cancer: Based on the comprehensive analysis of Annexins. J. Transl. Med..

[144] Lokman N.A., Ho R., Gunasegaran K., Bonner W.M., Oehler M.K., Ricciardelli C. (2019). Anti-tumour effects of all-trans retinoid acid on serous ovarian cancer. J. Exp. Clin. Cancer Res..

[145] Rawla P., Sunkara T., Gaduputi V. (2019). Epidemiology of pancreatic cancer: Global trends, etiology and risk factors. World J. Oncol..

[146] Gress T.M., Wallrapp C., Frohme M., Muller-Pillasch F., Lacher U., Friess H., Buchler M., Adler G., Hoheisel J.D. (1997). Identification of genes with specific expression in pancreatic cancer by cDNA representational difference analysis. Genes Chromosomes Cancer.

[147] Logsdon C.D., Simeone D.M., Binkley C., Arumugam T., Greenson J.K., Giordano T.J., Misek D.E., Kuick R., Hanash S. (2003). Molecular profiling of pancreatic adenocarcinoma and chronic pancreatitis identifies multiple genes differentially regulated in pancreatic cancer. Cancer Res..

[148] Karanjawala Z.E., Illei P.B., Ashfaq R., Infante J.R., Murphy K., Pandey A., Schulick R., Winter J., Sharma R., Maitra A. (2008). New markers of pancreatic cancer identified through differential gene expression analyses: Claudin 18 and annexin A8. Am. J. Surg. Pathol..

[149] Hata H., Tatemichi M., Nakadate T. (2014). Involvement of annexin A8 in the properties of pancreatic cancer. Mol. Carcinog..

[150] Pimiento J.M., Chen D.T., Centeno B.A., Davis-Yadley A.H., Husain K., Fulp W.J., Wang C., Zhang A., Malafa M.P. (2015). Annexin A8 is a prognostic marker and potential therapeutic target for pancreatic cancer. Pancreas.

[151] Inaguma S., Ito H., Riku M., Ikeda H., Kasai K. (2015). Addiction of pancreatic cancer cells to zinc-finger transcription factor ZIC2. Oncotarget.

[152] Inaguma S., Riku M., Ito H., Tsunoda T., Ikeda H., Kasai K. (2015). GLI1 orchestrates CXCR4/CXCR7 signaling to enhance migration and metastasis of breast cancer cells. Oncotarget.

[153] Deng P.C., Chen W.B., Cai H.H., An Y., Wu X.Q., Chen X.M., Sun D.L., Yang Y., Shi L.Q. (2019). LncRNA SNHG14 potentiates pancreatic cancer progression via modulation of annexin A2 expression by acting as a competing endogenous RNA for miR-613. J. Cell Mol. Med..

[154] Rawla P., Barsouk A. (2019). Epidemiology of gastric cancer: Global trends, risk factors and prevention. Prz. Gastroenterol..

[155] Ma F., Li X., Fang H., Jin Y., Sun Q. (2020). Prognostic value of ANXA8 in gastric carcinoma. J. Cancer.

[156] Yoo H.J., Yun B.R., Kwon J.H., Ahn H.S., Seol M.A., Lee M.J., Yu G.R., Yu H.C., Hong B., Choi K. (2009). Genetic and expression alterations in association with the sarcomatous change of cholangiocarcinoma cells. Exp. Mol. Med..

[157] Lee M.J., Yu G.R., Yoo H.J., Kim J.H., Yoon B.I., Choi Y.K., Kim D.G. (2009). ANXA8 down-regulation by EGF-FOXO4 signaling is involved in cell scattering and tumor metastasis of cholangiocarcinoma. Gastroenterology.

[158] Oka R., Nakashiro K., Goda H., Iwamoto K., Tokuzen N., Hamakawa H. (2016). Annexin A8 is a novel molecular marker for detecting lymph node metastasis in oral squamous cell carcinoma. Oncotarget.

[159] Staquicini D.I., Rangel R., Guzman-Rojas L., Staquicini F.I., Dobroff A.S., Tarleton C.A., Ozbun M.A., Kolonin M.G., Gelovani J.G., Marchio S. (2017). Intracellular targeting of annexin A2 inhibits tumor cell adhesion, migration, and in vivo grafting. Sci. Rep..

